# An Appendiceal Mucocele Presenting With Concurrent Gynecological Pathology: A Case Report

**DOI:** 10.7759/cureus.111176

**Published:** 2026-06-19

**Authors:** Gaushinee Vallimanalan, Pathma Rasa Annarasa

**Affiliations:** 1 General Surgery, Hospital Kuala Lumpur, Kuala Lumpur, MYS; 2 General Surgery, Columbia Asia Hospital, Seremban, MYS

**Keywords:** acute appendicitis, appendiceal mucocele, case report, pseudomyxoma peritonei, right iliac fossa pain

## Abstract

Appendiceal mucocele is a rare condition characterized by cystic distension of the appendix due to mucin accumulation, presenting a notable diagnostic dilemma that frequently mimics adnexal or ovarian masses in female patients. We describe a 25-year-old female who presented with right iliac fossa pain and was previously managed conservatively for an assumed appendiceal abscess, while concurrently undergoing active follow-up for a known ovarian cyst. Initial ultrasonography revealed a suspicious left adnexal mass, but subsequent computed tomography imaging demonstrated a fluid-filled, dilated appendix measuring 7.4 cm in length and 1.6 cm in maximum diameter, clearly exceeding the diagnostic threshold of 15 mm, alongside a thick-walled left ovarian cystic mass. The patient underwent a joint open laparotomy via a suprapubic transverse incision to address the dual pathology. Meticulous mobilization techniques using moist laparotomy pads were employed to prevent iatrogenic mucocele rupture. The appendiceal base was macroscopically free of disease, and an appendectomy was successfully performed alongside the gynecological resection. Histopathology confirmed a non-neoplastic appendiceal mucocele (retention cyst) and a left ovarian endometriotic cyst. The patient experienced an uneventful recovery and remains asymptomatic on outpatient follow-up. This case highlights the complexity of overlapping right lower quadrant pathologies and underscores the critical need for a high index of clinical suspicion and multidisciplinary surgical planning to ensure safe enucleation and prevent pseudomyxoma peritonei.

## Introduction

Appendiceal mucocele is a rare clinical entity, found in roughly 0.2% to 0.3% of all appendectomy specimens. It is important to note that "mucocele" is primarily a descriptive, macroscopic, and radiologic term reflecting the cystic dilation of the appendix by mucinous secretions. The underlying pathology can range from benign to malignant. The main histologic categories include simple or retention cysts, hyperplastic polyps, low-grade appendiceal mucinous neoplasms (LAMN), and mucinous adenocarcinoma. Previous reviews heavily emphasize that while "mucocele" is the preoperative radiologic diagnosis, the final surgical management and patient prognosis depend entirely on the definitive histologic subtype [1].

Clinically, these lesions are often asymptomatic or present with vague right lower quadrant pain. In female patients, appendiceal mucoceles have been noted in the literature for their striking ability to masquerade as adnexal or ovarian masses on imaging, a diagnostic overlap that frequently creates confusion during preoperative planning [2]. Here, we present a unique case of an appendiceal mucocele initially mimicking an adnexal mass in a 25-year-old patient with a prior diagnosis of an appendiceal abscess, highlighting the crucial need for multidisciplinary coordination and a high index of clinical suspicion. The purpose of this manuscript is to highlight the unusual clinical presentation of a benign appendiceal mucocele masquerading as a gynecological pathology, providing a clear diagnostic strategy to prevent misdiagnosis and avoid devastating intraoperative rupture.

## Case presentation

A 25-year-old female presented to the emergency department of a private hospital in Seremban, Malaysia, reporting a two-day history of right iliac fossa pain, accompanied by a noticeable loss of appetite and dysuria. She remained afebrile and denied experiencing nausea or vomiting. Her medical history was significant for a hospitalization one month prior at another institution, where she was diagnosed with an appendiceal abscess based on clinical presentation and ultrasound features, and successfully managed conservatively with intravenous antibiotics. Notably, given her current presentation, it is highly probable that this earlier diagnosis actually represented an inflamed mucocele that was misinterpreted on ultrasound. Concurrently, she was under active follow-up with the gynecology team for a known ovarian cyst.

Physical examination revealed a patient who was afebrile but tachycardic, with a pulse rate of 100 beats per minute. Abdominal evaluation demonstrated localized tenderness over the right iliac fossa, complemented by localized guarding and rebound tenderness. Initial laboratory investigations, including a total white blood cell count and a urine full examination and microscopic examination (FEME), were unremarkable, though her C-reactive protein (CRP) level was significantly elevated at 25 mg/L (normal reference range: <5 mg/L for adult females).

A preliminary abdominal ultrasound identified a left adnexal cyst measuring 3.4 x 4.0 x 2.3 cm. To further delineate the pathology, a computed tomography (CT) scan was performed. The scan revealed an elongated, fluid-filled, and dilated appendix measuring 7.4 cm in length and 1.6 cm at its maximum diameter. This appendiceal diameter clearly exceeds the established >15 mm radiological threshold suggestive of a mucocele [2]. Contiguous with the appendix was a cystic dilation within the cecum measuring 2.7 x 3.2 cm. Additionally, a thick-walled left ovarian cystic mass measuring 4.3 x 6.1 x 4.5 cm was noted (Figure 1).

**Figure 1:**
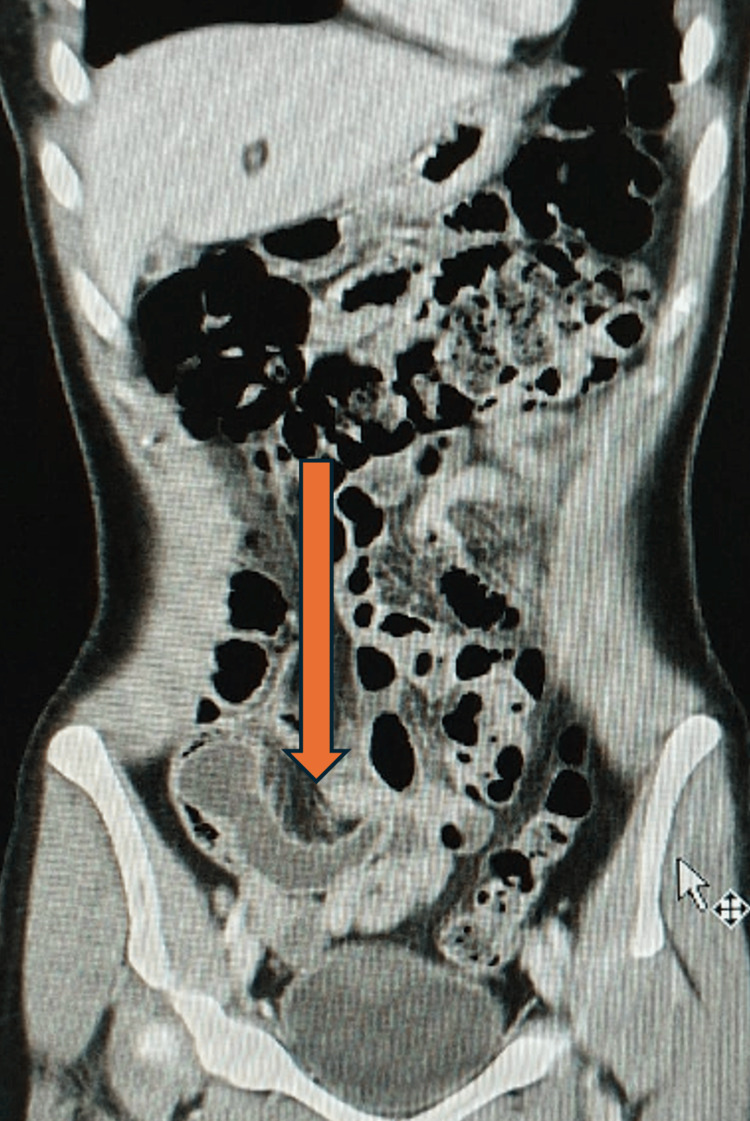
CT scan findings showing a dilated, fluid-filled, and elongated appendix and cystic dilation within the cecum.

A provisional diagnosis of acute appendicitis complicated by an appendiceal mucocele and a concurrent right ovarian cyst was established. Given the dual pathology, a joint operation with the gynecology team was scheduled for the following day.

The surgical teams utilized an open approach via a suprapubic transverse incision. This specific incision was chosen over a standard midline approach to optimize exposure for the concurrent gynecologic resection while ensuring a superior cosmetic outcome. Intraoperative frozen section analysis was not available at our center. Intraoperative exploration confirmed an inflamed appendix featuring a mucocele that extended into the cecum (Figures 2, 3). To avoid iatrogenic rupture and the catastrophic spillage of mucin, meticulous mobilization of the cecum and appendix was performed using gentle traction with moist laparotomy pads, ensuring instruments were kept strictly off the fragile body of the mucocele [3,4]. A careful inspection of the abdomen revealed no mesoappendiceal nodes or peritoneal nodules.

**Figure 2:**
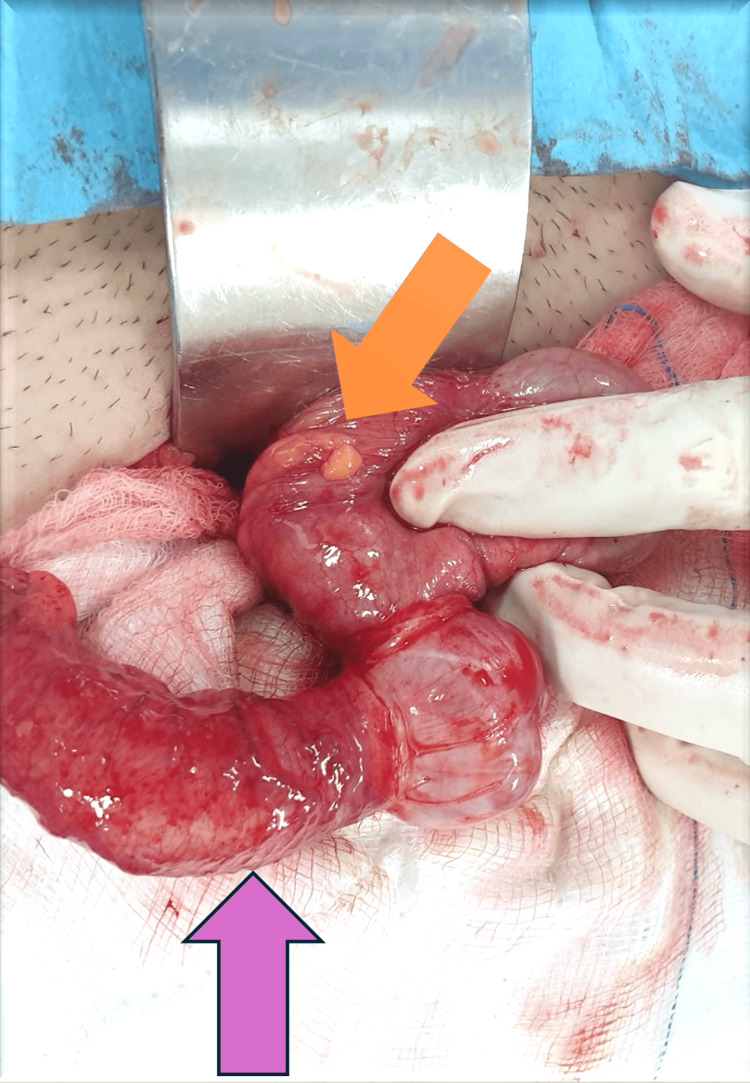
Intraoperative view showing the inflamed appendix featuring a mucocele extending into the cecum. The orange arrow points precisely to the cecum, and the purple arrow points at the appendix.

**Figure 3:**
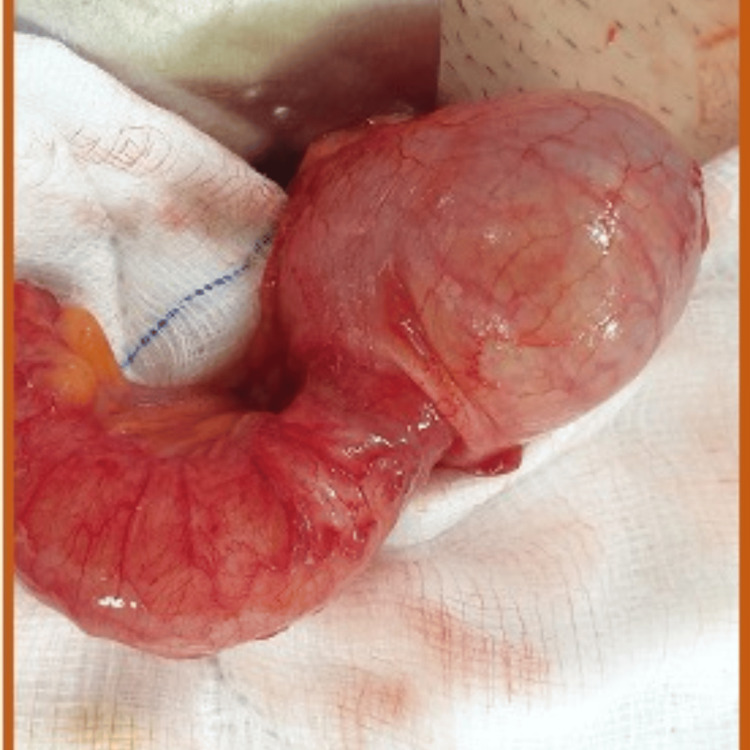
A closer, magnified intraoperative view detailing the appendiceal mucocele.

The surgical team successfully shelled out the mucocele from the cecum via an incision precisely at the appendiceal base, which was macroscopically free of disease. Notably, there was no continuous mucosal tract between the cecum and the appendix; both mucosal layers were naturally sealed. The cecal serosa was meticulously repaired utilizing interrupted sero-submucosal sutures with Polyglactin 3-0. The resected appendix measured 80 mm in length with a 20 mm diameter, exhibiting a 3.0 cm mucus-filled cyst at its apex (Figure 4).

**Figure 4:**
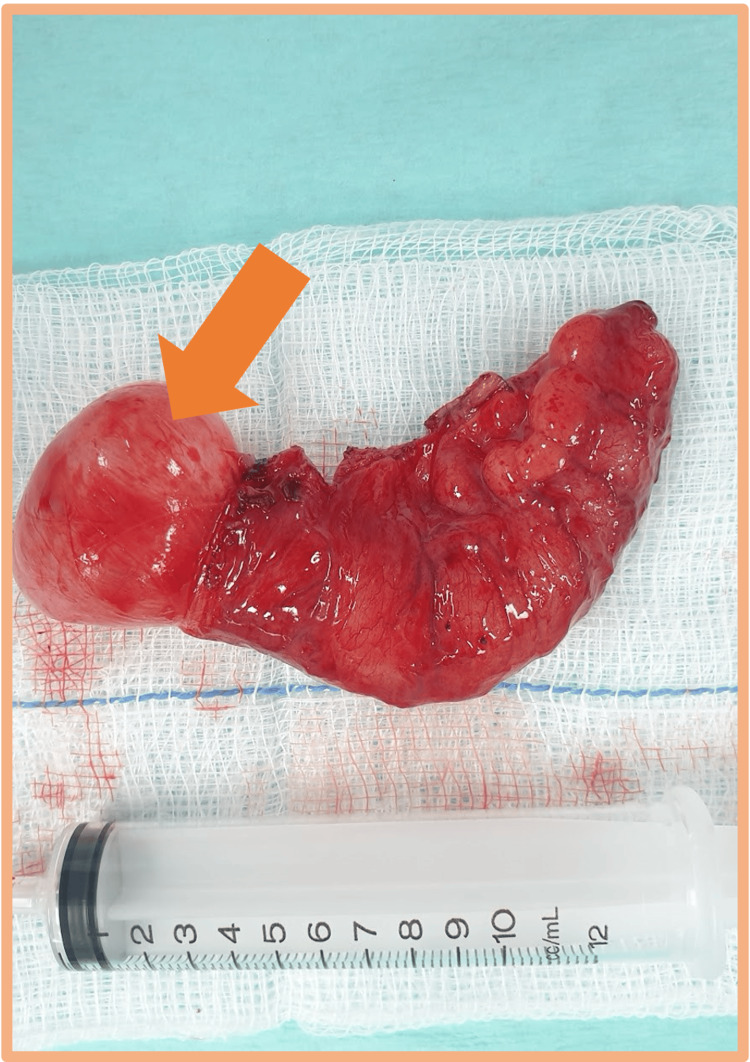
Gross specimen of the resected appendix measuring 80 mm in length, demonstrating a prominent 3.0 cm mucus-filled cystic dilation (mucocele) at the apex.

The postoperative phase was entirely uneventful, permitting the patient's discharge the subsequent day.

Histopathological evaluation of the appendix displayed an intact mucosa lined by a single layer of columnar epithelium. The appendiceal wall and serosa demonstrated mild, chronic inflammatory cell infiltrates alongside congested serosal blood vessels. The apical mucocele was filled with mucus, featuring a denuded lining epithelium. Crucially, the lesion was confirmed to be a non-neoplastic mucocele (retention cyst); there was no epithelial atypia, no mucin dissection into the appendiceal wall, and no extra-appendiceal mucin identified. The final diagnosis was acute appendicitis with a benign mucocele, and the left ovarian mass was confirmed to be an endometriotic cyst. During her outpatient clinic follow-up at six months, conducted as per CARE (CAse REport) guidelines [5], she reported excellent clinical recovery with no evidence of recurrence.

## Discussion

The preoperative diagnosis of an appendiceal mucocele remains notoriously difficult due to its non-specific clinical picture. Our case is particularly unique because the patient presented with right iliac fossa pain and a recent history of a conservatively managed "appendiceal abscess," while concurrently undergoing follow-up for a known ovarian cyst. This diagnostic overlap and frequent confusion with adnexal masses are well-documented in radiological literature, underscoring the necessity for high-resolution CT imaging when ultrasound yields ambiguous results or multiple pathologies are present in female patients [2,6].

Comparing our experience with similar literature highlights the value of multidisciplinary surgical planning. When gynecologic pathology and an appendiceal mass present concurrently, the surgical approach must be carefully tailored. In our case, the choice of an open laparotomy via a suprapubic transverse incision was deliberately driven by the need for dual surgical exposure, allowing both the gynecology team and general surgery team to operate safely without compromising the mucocele's integrity [7,8].

It is absolutely critical to distinguish between the management of non-neoplastic mucoceles and neoplastic lesions. Neoplastic mucoceles, particularly LAMN and mucinous adenocarcinomas, carry a severe risk of progressing to pseudomyxoma peritonei (PMP) if they rupture. PMP is characterized by extensive mucinous ascites and widespread peritoneal implants, a condition that requires highly aggressive management, typically involving cytoreductive surgery combined with heated intraperitoneal chemotherapy (HIPEC) [9,10]. Conversely, non-neoplastic mucoceles, such as the retention cyst seen in our patient, do not exhibit epithelial atypia or wall dissection and are entirely curative with a careful appendectomy alone, provided no spillage occurs [11-13].

Limitations

We must acknowledge several limitations inherent to this report. As a single case presentation, the specific clinical pathway described may not be universally applicable to all populations. Furthermore, our surgical management was limited by the absence of intraoperative frozen section capabilities, which, if available, could have helped definitively guide the extent of resection in real-time. Finally, while the patient is currently disease-free, the postoperative follow-up period remains relatively short.

## Conclusions

Appendiceal mucoceles present a significant diagnostic challenge, particularly when their presentation is confounded by concurrent adnexal pathology in female patients. This unique case, initially managed as a simple abscess and complicated by a concurrent ovarian cyst, emphasizes the critical need for a high index of suspicion and multidisciplinary surgical planning. Precise, gentle intraoperative handling is paramount to preventing mucocele rupture and the devastating consequence of pseudomyxoma peritonei. Ultimately, identifying the precise histologic subtype remains the cornerstone for determining patient prognosis and guiding appropriate long-term management. This unique diagnostic trajectory provides crucial educational value to clinicians, demonstrating that persistent right lower quadrant masses, especially those with a history of recurrent or conservatively managed "abscesses," warrant a high clinical suspicion for an underlying appendiceal mucocele.
